# Orexins in apoptosis: a dual regulatory role

**DOI:** 10.3389/fncel.2024.1336145

**Published:** 2024-04-12

**Authors:** Simona Cavalu, Sameh Saber, Rabab S. Hamad, Mustafa Ahmed Abdel-Reheim, Elsayed A. Elmorsy, Mahmoud E. Youssef

**Affiliations:** ^1^Faculty of Medicine and Pharmacy, University of Oradea, Oradea, Romania; ^2^Department of Pharmacology, Faculty of Pharmacy, Delta University for Science and Technology, Gamasa, Egypt; ^3^Biological Sciences Department, College of Science, King Faisal University, Al Ahsa, Saudi Arabia; ^4^Central Laboratory, Theodor Bilharz Research Institute, Giza, Egypt; ^5^Department of Pharmaceutical Sciences, College of Pharmacy, Shaqra University, Shaqra, Saudi Arabia; ^6^Department of Pharmacology and Toxicology, Faculty of Pharmacy, Beni-Suef University, Beni Suef, Egypt; ^7^Department of Pharmacology and Therapeutics, College of Medicine, Qassim University, Buraidah, Saudi Arabia; ^8^Department of Clinical Pharmacology, Faculty of Medicine, Mansoura University, Mansoura, Egypt

**Keywords:** orexins, apoptosis, CNS disorders, neuroprotection, cell signaling, new therapeutic target

## Abstract

The orexins, also referred to as hypocretins, are neuropeptides that originate from the lateral hypothalamus (LH) region of the brain. They are composed of two small peptides, orexin-A, and orexin-B, which are broadly distributed throughout the central and peripheral nervous systems. Orexins are recognized to regulate diverse functions, involving energy homeostasis, the sleep-wake cycle, stress responses, and reward-seeking behaviors. Additionally, it is suggested that orexin-A deficiency is linked to sleepiness and narcolepsy. The orexins bind to their respective receptors, the orexin receptor type 1 (OX1R) and type 2 (OX2R), and activate different signaling pathways, which results in the mediation of various physiological functions. Orexin receptors are widely expressed in different parts of the body, including the skin, muscles, lungs, and bone marrow. The expression levels of orexins and their receptors play a crucial role in apoptosis, which makes them a potential target for clinical treatment of various disorders. This article delves into the significance of orexins and orexin receptors in the process of apoptosis, highlighting their expression levels and their potential contributions to different diseases. The article offers an overview of the existing understanding of the orexin/receptor system and how it influences the regulation of apoptosis.

## 1 Introduction

Orexins, alternatively referred to as hypocretins, are chemical messengers generated within limited clusters of neurons found in the lateral hypothalamic (LH) and perifornical hypothalamic regions (PFA). The term “orexin” is derived from the Greek word “orexis,” which translates to hunger or appetite. The primary role of orexins lies in controlling wakefulness, alertness, food consumption, and behaviors associated with rewards by affecting specific clusters of brain nuclei (Sakurai, 2014).

**Figure 1:**
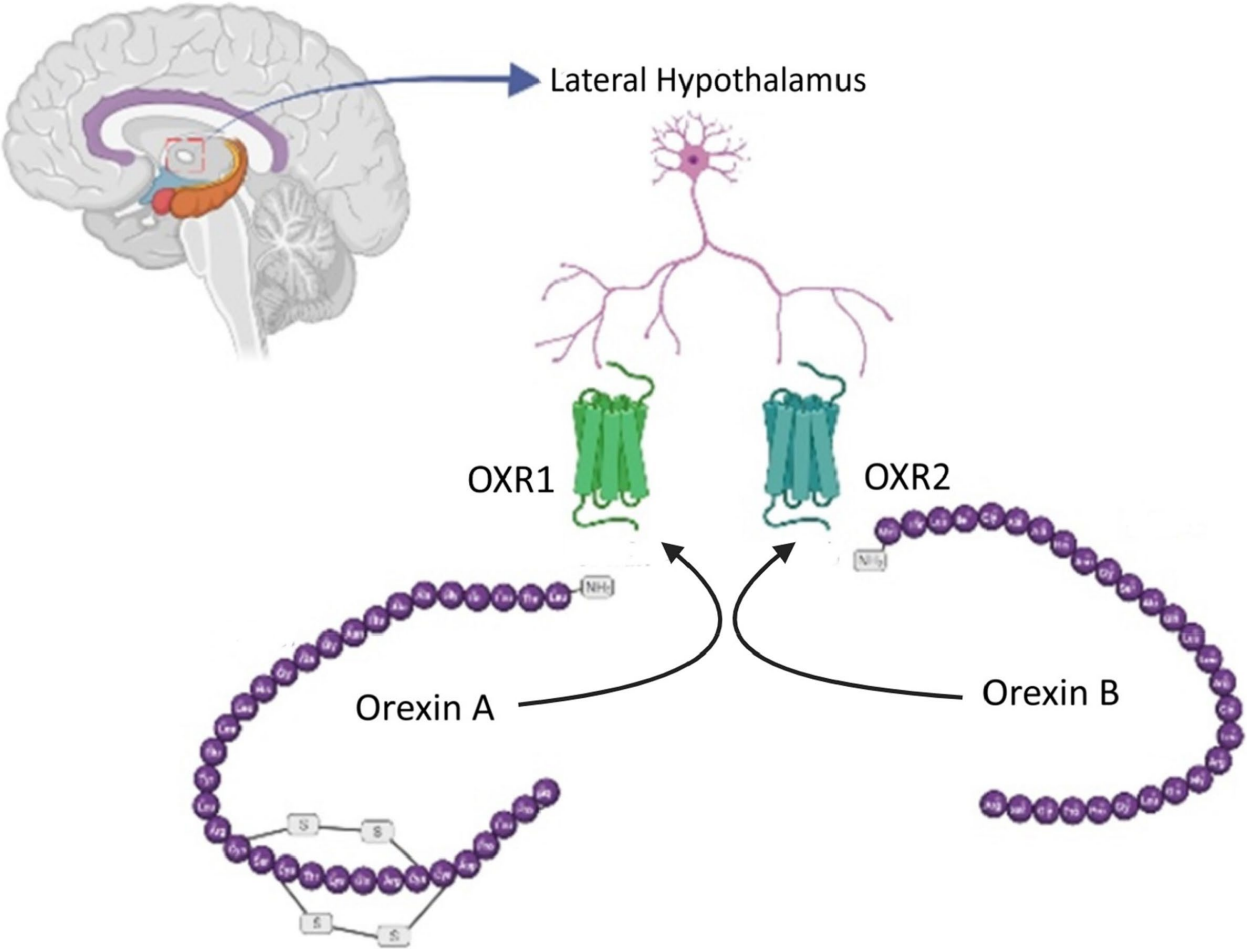
Orexin neurotransmitters. Two orexin peptides, orexin-A (33 amino acids) and orexin-B (28 amino acids), are generated from preproorexin cleavage. Orexin-A binds to both OX1R and, to a lesser extent, OX2R, while orexin-B exhibits a higher affinity for OX2R.

There are two forms of orexin peptides, both derived from the cleavage of preproorexin (Karteris et al., 2004). These are orexin-A, which is made up of 33 amino acids, and orexin-B, which is composed of 28 amino acids (Figure 1). Orexin-A can bind to both the orexin-1 receptor (OX1R) and, to a lesser extent, the orexin-2 receptor (OX2R), while orexin-B has a higher binding affinity for OX2R (Lang et al., 2004). Despite the widespread distribution of these receptors, research into orexin signaling has primarily focused on OX1R owing to the lack of an effective and available OX2R antagonist (Wang et al., 2018).

**Figure 2:**
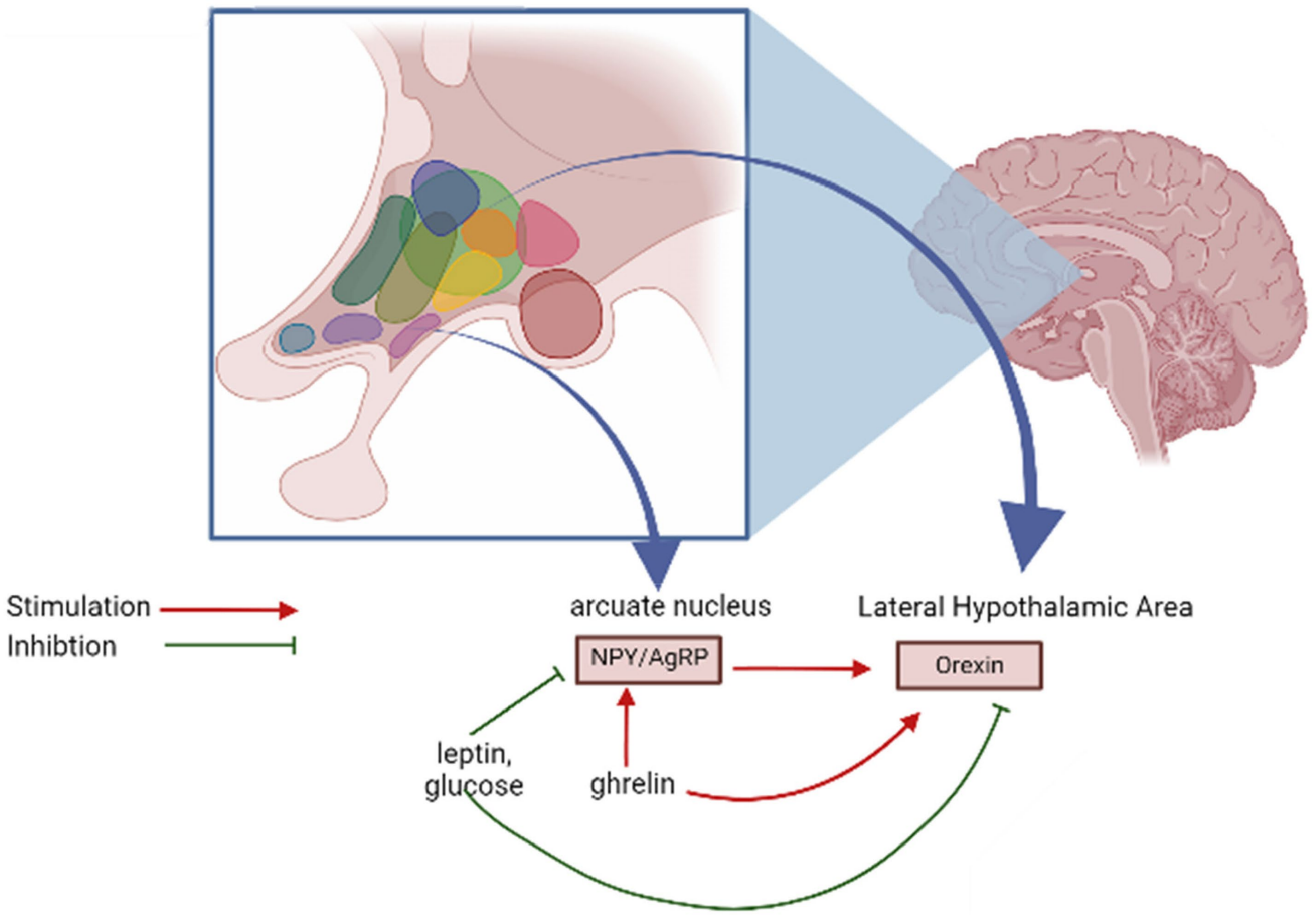
Regulation of orexin release.

Neurons synthesizing orexins establish bidirectional connections with mediobasal hypothalamic structures responsible for managing food consumption (Sakurai, 1999). There is supporting evidence indicating that orexin neurons in the lateral hypothalamus (LH) can be considered “second-order” neurons, as they receive signals from neurons within the arcuate nucleus that express neuropeptide Y (NPY) and agouti-related peptide (AgRP) (Berthoud, 2002). This suggests that these neurons are involved in the integration processes that facilitate and promote food consumption (Reichelt et al., 2015). Nonetheless, there is also supporting evidence indicating that orexin neurons can function as “first-order” neurons, directly responding to metabolic cues such as leptin, glucose, and ghrelin. These neurons seem to exhibit characteristics of both first- and second-order neurons, participating in the intricate integration of signals from neuropeptides and adipostatic factors that govern feeding behavior (Adamantidis and De Lecea, 2009) (Figure 2).

Concerning feeding behavior, extensive documentation exists on the appetite-stimulating effects of orexin-A regarding food intake (Öztürk et al., 2022). Administering orexin-A directly into the cerebral ventricles has been demonstrated to boost food consumption in rodents. Moreover, in experiments where rats have options, they tend to preferentially increase their consumption of favored diets, particularly those rich in saturated fat, when exposed to orexin-A (Yamanaka et al., 1999). Blocking the orexin-A-induced increase in appetite and behavioral satisfaction has proven effective through the antagonism of the OX1R (orexin receptor 1) (White et al., 2005).

Orexin neurons form bidirectional connections with regions in the mediobasal hypothalamus that play a role in regulating food intake. New studies suggest that orexin neurons located in the lateral hypothalamus (LH) can function as “second-order” neurons, receiving input from neurons containing neuropeptide Y (NPY) and Agouti-related peptide (AgRP) situated within the arcuate nucleus. This suggests their involvement in the process of integrating signals related to food intake. Conversely, these orexin neurons can also function as “first-order” neurons, directly responding to metabolic signals such as leptin, glucose, and ghrelin. The intricacy of their functions likely encompasses both first- and second-order characteristics in the complex integration of signals from neuropeptides and signals related to adipostasis, which collectively govern feeding behavior.

## 2 Orexin receptors

In mammals, there exist two distinct types of orexin receptors, explicitly the OX1R and the OX2R (Nitkiewicz et al., 2010). Both of these receptors belong to class A of G-protein-coupled receptors (GPCRs) (Yang et al., 2021). Interestingly, OX1R is exclusive to mammals and is agreed to have developed from the original OX2R via gene duplication events that occurred throughout the early stages of mammalian evolution (Soya and Sakurai, 2020). Conversely, OX2R exists in all vertebrates, implying that it represents the ancestral form of orexin receptors (Tam et al., 2011). Due to its relatively recent phylogenetic origin compared to OX2R, OX1R appears to be involved in more intricate and complex physiological functions (Zhang et al., 2013). Research has indicated that mice lacking OX1R display behavior resembling anxiety (Demidova et al., 2022). Furthermore, it has been observed that OX1R within the locus coeruleus (LC) noradrenaline neurons contribute to the expression and consolidation of conditioned fear memory. This is achieved through the stimulation of LC neurons, which project to the lateral amygdala. This pathway also acts a role in the generalization of fear memory (Kaplan et al., 2022). OX1R has also been demonstrated to participate in heightened responses to conditioned cues, activating motivational responses in rats and influencing feeding behavior associated with reward (Choi et al., 2010). These results indicate that OX1R has a part to play in the emotional and motivational processes observed in mammals (Sakurai, 2014).

## 3 Molecular mechanisms of orexin/receptor interaction

Orexins are neuropeptides that play a key role in regulating various physiological functions, such as arousal, appetite, energy homeostasis, and stress response (Grafe and Bhatnagar, 2018). Orexins bind to two types of GPCRs, OX1R and OX2R, which have distinct but overlapping expression patterns and signaling pathways in the brain and peripheral tissues (Wang et al., 2018).

Understanding the molecular mechanisms of orexin interaction with receptors is essential for explaining the physiological and pathological roles of orexins and developing novel therapeutics for orexin-related disorders. Orexin A is composed of 33 amino acids, while orexin B contains 28 amino acids. They share a common C-terminal region that is crucial for receptor binding. Orexins adopt an alpha-helical conformation in solution and in complex with receptors, as revealed by nuclear magnetic resonance (NMR) and X-ray crystallography research (Yin et al., 2016). Orexins bind to the extracellular domains of OX1R and OX2R, which consist of seven transmembrane helices connected by three extracellular loops and an N-terminal tail (Vu et al., 2021).

The molecular interactions between orexins and receptors involve multiple contacts between the C-terminal region of orexins and the N-terminal tail, the first extracellular loop, and the second extracellular loop of receptors, The C-terminal region of orexins forms a hydrophobic core that interacts with a conserved pocket formed by the N-terminal tail and the first extracellular loop of receptors (Takai et al., 2006). The C-terminal region also contains a polar residue (Asn or Asp) that forms a hydrogen bond with a conserved residue (Tyr or His) in the second extracellular loop of receptors (Karhu et al., 2015). These interactions are essential for orexin binding and receptor activation. In addition, the N-terminal region of orexins contributes to receptor binding and selectivity by forming specific contacts with residues in the N-terminal tail and the first extracellular loop of receptors (Karhu et al., 2019). The N-terminal region also modulates the conformational changes of receptors upon ligand binding, which affects the coupling efficiency of receptors to G proteins.

The binding of orexins to OX1R and OX2R induces conformational changes in the intracellular domains of receptors, which enable the interaction with various G proteins and downstream effectors (Couvineau et al., 2019). OX1R preferentially couples to Gq/11 proteins, which stimulate phospholipase C (PLC) and enhance intracellular calcium levels (Berrendero et al., 2018). OX2R couples to both Gq/11 and Gi/o proteins, which prevent adenylyl cyclase (AC) and decrease cyclic adenosine monophosphate (cAMP) (Urbańska et al., 2012). Both OX1R and OX2R can also couple to Gs proteins, which activate AC and increase cAMP levels. The differential coupling of OX1R and OX2R to G proteins results in distinct cellular responses to orexins in diverse tissues and cell types.

## 4 Physiological effects of orexins

The role of orexins extends beyond the central nervous system to the periphery. Orexin receptors are expressed not only in the brain, but also in various peripheral tissues, such as the gastrointestinal tract, kidneys, gonads, pancreas, and adrenal glands (Kirchgessner and Liu, 1999; Randeva et al., 2001; Nakabayashi et al., 2003; Liguori et al., 2018; Couvineau et al., 2019) (Table 1). These peripheral effects of orexins have been investigated in both animals and humans and have known to play important roles in regulating various physiological processes such as glucose homeostasis, energy balance, and cardiovascular function. Studies have shown that the orexin system plays a role in regulating glucose metabolism and insulin sensitivity in peripheral tissues, particularly in the liver (Greene et al., 2020). Orexin A has been shown to have insulin-sensitizing effects and to reduce liver glucose production. Moreover, the orexin system has been implicated in regulating body weight, body fat distribution, and metabolism. Orexin-A has been revealed to reduce body weight and improve insulin sensitivity in obese animals (Zarifkar et al., 2017). The orexin system has been shown to play a role in regulating inflammation, both in the central nervous system and in peripheral tissues (Couvineau et al., 2019).

**Table 1:** Physiological effects of orexin.

Organ	Effect of orexin	References
Brain	Regulates various physiological processes	Couvineau et al. (2019)
Gastrointestinal tract	Increases gastric motility, promotes enzyme release	Baccari (2010)
Kidneys	Not specified	
Gonads	Not specified	
Pancreas	Stimulates insulin secretion, regulates glucose homeostasis	Nowak et al. (2005)
Adrenal glands	Not specified	
Liver	Reduces liver glucose production, insulin sensitivity	Greene et al. (2020)
Peripheral tissues (general)	Regulates glucose metabolism, insulin sensitivity	Zarifkar et al. (2017)
Adipocytes	Modulates adipocyte function	Suarez et al. (2020)
Cardiovascular system	Regulates blood pressure, heart rate	Carrive (2013)
Stress response (hypothalamic–pituitary–adrenal axis)	Modulates stress response, regulates CRH release	Spinazzi et al. (2006)
Immune system	Implication for the management of inflammatory and autoimmune conditions	Becquet et al. (2019)

In the gastrointestinal tract, orexins have been shown to increase gastric motility and promote the release of digestive enzymes, thereby increasing nutrient absorption (Baccari, 2010). In the pancreas, orexins have shown to stimulate the secretion of insulin and regulate glucose homeostasis (Nowak et al., 2005). Orexins also play a role in the regulation of energy balance by modulating the function of adipocytes and regulating the release of hunger-inducing hormones such as ghrelin (Suarez et al., 2020). In addition, orexins have shown to play a role in cardiovascular function by regulating blood pressure and heart rate (Carrive, 2013). Orexins have additionally been associated with the control of the stress response by influencing the functioning of the hypothalamic-pituitary-adrenal (HPA) axis and the release of CRH from the hypothalamus (Spinazzi et al., 2006).

Orexin is integral to the immune system, engaging with its receptors found on immune cells. Research indicates the presence of orexin receptors, specifically OX1R and OX2R, across a range of immune-related tissues and cells, such as T lymphocytes and myeloid cells, which points to a direct influence of orexins (Becquet et al., 2019). Treatment with Orexin A has been observed to alter the population of immune cells, potentially leading to immunosuppressive outcomes in specific cell types, including granulocytic and monocytic myeloid-derived suppressor cells. Furthermore, Orexin A has demonstrated sustained positive effects in managing autoimmune disorders, such as experimental autoimmune encephalomyelitis, showcasing its capacity to influence neuroinflammatory pathways (Becquet et al., 2019). In essence, the involvement of orexin within the immune system underscores its regulatory function and suggests significant implications for the management of inflammatory and autoimmune conditions.

## 5 Apoptosis

Apoptosis is a programmed cell death that is believed to be vital in keeping the stability of cell populations within an organism. It is a well-controlled process that takes place due to various internal and external stimuli and is crucial for the normal functioning of cells and tissues. The term “apoptosis” was first used to describe this process due to the characteristic shrinkage and fragmentation of cells into smaller units known as apoptotic bodies (Elmore, 2007). Unlike apoptosis, necrosis is another type of cell death that occurs as a result of cellular injury from external factors such as toxins or trauma. Necrosis is an uncontrolled process that can cause inflammation and harm to tissue, whereas apoptosis is a regulated process that does not result in inflammation or tissue damage (Fink and Cookson, 2005).

Apoptosis is triggered by signals received by the cell, including DNA damage, growth factor withdrawal, oxidative stress, and viral infections. These signals activate specific enzymes called caspases, which cause the degradation of cellular proteins, resulting in cell shrinkage, chromatin condensation, and fragmentation into apoptotic bodies (Jan and Chaudhry, 2019). This process of apoptosis is crucial for preserving the balance of cell populations in an organism. For instance, it plays a critical role in the development of the nervous system by selectively eliminating excess neurons to form functional neural circuits (Hollville et al., 2019). Additionally, it also helps in eliminating damaged or abnormal cells, thus avoiding the development of cancer and other diseases.

The molecular mechanisms that control apoptosis are complex and involve the triggering of specific signaling pathways and the interaction of many different proteins. The two main pathways that control apoptosis are the extrinsic pathway, which is activated by signals from outside the cell, and the intrinsic pathway, which is activated by signals within the cell (Elmore, 2007). Apoptosis is regulated by a delicate balance between pro-apoptotic and anti-apoptotic signals. Pro-apoptotic signals promote cell death, while anti-apoptotic signals inhibit cell death. Disruptions in this balance can result in a failure of apoptosis, which can contribute to the development of cancer and other diseases (Pistritto et al., 2016). Cancer cells often exhibit resistance to apoptosis, which allows them to proliferate uncontrollably. In addition, abnormal apoptosis has been implicated in numerous diseases, involving neurodegenerative disorders, autoimmune diseases, and cardiovascular disease (Ghavami et al., 2014). Because of its central role in regulating cell death, apoptosis is a critical target for the development of new therapies. For example, drugs that can enhance apoptosis in cancer cells have been developed and are being investigated in clinical trials. Conversely, drugs that can inhibit apoptosis in certain diseases, such as neurodegenerative disorders, are also being developed. The apoptotic process involves various cellular organelles like mitochondria, Golgi apparatus, lysosomes, endoplasmic reticulum, centrosomes, cell membrane, and nucleus.

## 6 Effect of orexins on caspases

The initiation of apoptosis and the triggering of proapoptotic proteins lead to the triggering of caspases. These proteases are accountable for the precise cleavage of particular protein substrates and hold considerable importance in controlling whether cells survive or undergo programmed cell death. Caspases also contribute significantly to various other cellular processes, embracing inflammation, cell proliferation, and differentiation (Shalini et al., 2015). Currently, 11 human caspases have been identified and can be separated into two groups: inflammatory caspases (caspase 1, 4, 5) and apoptotic caspases, which are further divided into initiator caspases (caspases 2, 8, 9, and 10) and executive caspases (caspases 3, 6, and 7) (Van Opdenbosch and Lamkanfi, 2019).

Cell demise initiated by Orexin A exhibited characteristics such as condensed chromatin structure, which is tightly packed, and it necessitated the activation of new gene transcription and protein synthesis, both of which are well-recognized indicators of programmed cell death. This phenomenon might also be linked to caspase activation alongside various other apoptotic pathways (Ammoun et al., 2006). In a similar vein, previous findings had proposed that the activation of orexin receptors had the potential to trigger apoptosis in rat C6 glioma cells by stimulating the caspase signaling pathways (Biegańska et al., 2012). In a separate investigation, Orexins were found to induce cell death through apoptosis, relying on caspase activity, and led to significant inhibition of cell growth within Chinese hamster ovary cells that had been transfected with OX2R cDNA. This effect was achieved by activating caspase 3 and caspase 9 (Voisin et al., 2006). The cell death induced by orexin-A exhibited features such as condensed chromatin, a tightly packed structure. Furthermore, it necessitated the initiation of new gene transcription and protein synthesis, which are well-established characteristics of programmed cell death. Additionally, this phenomenon might be connected to the activation of caspases, alongside other mechanisms associated with apoptosis (Ammoun et al., 2006).

Orexin receptor presence was detected in various cancer models. Orexins did not influence cell multiplication but instead facilitated cell death, as evidenced by observable alterations in cell structure, fragmentation of DNA, condensation of chromatin, release of cytochrome c into the cytosol, and activation of caspase-3 and caspase-7 enzymes. The occurrence of OX1R and the orexins’ pro-cell death effects were evident in colon cancer cell lines such as Caco-2, SW480, and LoVo, but notably absent in typical colonic epithelial cells (Rouet-Benzineb et al., 2004). In colon cancer cell lines, Orexin acted as an intrinsic pro-apoptotic peptide by triggering the activation of OX1R (Rouet-Benzineb et al., 2004; Laburthe and Voisin, 2012). When treating chemoresistant pancreatic tumors, which had developed resistance to Nab-paclitaxel or gemcitabine through succeeding mice xenografts, it was discovered that orexin A exhibited a remarkable capability to significantly impede tumor growth (Voisin et al., 2022). This effect was achieved by boosting apoptosis mediated by caspase 3 and 7. Importantly, no resistance to orexin-A was observed in these tumors (Voisin et al., 2022). The caspase-mediated apoptosis process is generally governed by two primary pathways: the extrinsic pathway, regulated by caspase-8 and initiated through death receptors, and the intrinsic pathway, governed by caspase-9 and initiated within the mitochondria (Wen et al., 2012). Caspase-3, functioning as the downstream executor, facilitates the cleavage of cellular target proteins, ultimately leading to cell death. Research has provided evidence indicating that orexins can induce apoptosis and hinder cell proliferation in both human and rat pancreatic tumor cells and CHO cells. These effects are achieved via the stimulation of caspase-3 activity (Chen L. et al., 2013; Dayot et al., 2018) (Table 2).

**Table 2:** The effects of orexin on different molecular pathways of apoptosis.

Target	Specific proteins targeted	Effects of orexin	References
Effect on caspases	Caspase-3, Caspase-7, Caspase-9	Promotes apoptosis by inducing Caspase-3, Caspase-7, and Caspase-9	Voisin et al. (2022)
	Cytochrome C	Suppresses cleaved caspase-9 and caspase-3	Suo et al. (2018)
		Cytochrome c release	Xu et al. (2021b)
		Inhibits cytochrome C expression	Xu et al. (2021b)
		Stimulation of the enzyme phosphotyrosine phosphatase SHP-2, followed by the initiation of mitochondrial apoptosis mediated by cytochrome c	Marcos and Coveñas (2023)
Mitochondrial mechanism of apoptosis	Bcl-2, Bax	Increases Bcl-2 expression Decreases Bax expression Raise Bcl-2/Bax ratio	Safdar et al. (2021)
Effect of orexins on receptor-related mechanism of apoptosis	TNF-α	Reduce TNF-α-mediated inflammation Anti-apoptotic effect	Messal et al. (2018)
	Fas receptors	Reduces inflammation Potentially exhibits antiapoptotic effects	Dondelinger et al. (2016)
		Provides insights into chemotherapeutic intervention challenges with Fas ligand-mediated apoptosis	Wolpin and Mayer (2008), Segal and Saltz (2009), Voisin et al. (2011)
Endoplasmic reticulum stress and apoptosis	GRp87 IRE1α XBP1 PERK Unfolded Proteins, mTOR-XBP1	Prevents ER stress and inflammation	Tsuneki et al. (2022)
	Ca2+	At higher concentrations, orexins increase Ca2+ release from the endoplasmic reticulum and increase apoptosis	Xia et al. (2009)
Modulation of p53 activity	p53	Modulates p53 activity, leading to inhibition of apoptosis	Sokołowska et al. (2014)
Regulation of Caspase-2	Caspase-2	Reduces caspase-2 activity	Sokołowska et al. (2014)

Conversely, orexins have been observed to promote the proliferation of neuronal cells by inhibiting the activity of caspase-3. Apaf-1 (Apoptotic protease activating factor 1) acts as an activator of caspase-3 in the presence of cytochrome C. Interestingly, Apaf-1 expression was found to be absent in normal neuronal cells, potentially conferring resistance to apoptosis induced by cytochrome c in these cells (Shakeri et al., 2021). This lack of Apaf-1 expression in normal neural tissues renders them resistant to the apoptotic effects instigated by cytochrome c. This underscores the pivotal role of Apaf-1 in mediating apoptosis specifically in brain tumors, while sparing normal neuronal cells. Furthermore, research has found that treatment with orexin A can significantly inhibit apoptosis induced by serum starvation, which was endorsed by reduced activity of caspase-9 (Liu et al., 2015). However, under the same conditions, orexin A had no impact on the activity of caspase-8, indicating that it can restrain the intrinsic apoptotic pathway and guard cells from undergoing apoptosis (Liu et al., 2015) (Table 2).

Other studies have supported these findings, indicating that orexin receptor activation reduces caspase activity and cellular apoptosis. In SGC-7901 gastric cancer cells, The expression of OX1R was observed and orexin A stimulated SGC-7901 cell proliferation and sustainability, diminished the pro-apoptotic activity of caspase-9, and guarded the cells from apoptosis in a dose-dependent manner (Liu et al., 2015). In a separate investigation, it was found that orexin A had an inhibitory effect on apoptosis in gastric cancer cells, and this inhibition was mediated through OX1R via the protein kinase B (Akt) pathway (Wen et al., 2015). Furthermore, it has been uncovered that the stimulation of OX1R may play a critical role in the development of pancreatic cancer and could serve as a promising therapeutic target for individuals with pancreatic cancer. This potential treatment avenue involves the modulation of the Akt/mTOR pathway alongside the stimulation of caspase 3 and 9 (Chen Y. et al., 2013; Suo et al., 2018).

## 7 Effect of orexins on mitochondrial mechanism of apoptosis

The significance of mitochondria in the process of apoptotic cell death is considerable and can be initiated by a range of different stimuli. A crucial stage within this series of events revolves around the release of cytochrome c and other proteins from the interior of mitochondria into the adjacent cytosol. This occurrence is a consequence of heightened permeability in the membranes of the mitochondria (Wang and Youle, 2009). Cytochrome c plays a pivotal role by serving as a foundation for the assembly of a structure known as the apoptosome. This apoptosome consists of both oligomeric proteins and procaspases-9 molecules. After activation, caspase-9 dissociates from the apoptosome and oversees the activation of other procaspase-9 molecules. This, in turn, triggers the activation of effector caspases, including caspase-3, -6, and -7 (Iordanov et al., 2005).

Mitochondria’s role in controlling and enhancing the apoptotic pathway is facilitated by the Bcl-2 protein family. Back in 1988, Bcl-2 was primarily acknowledged as a gene product emerging from translocations, a prevalent genetic characteristic frequently observed in B-cell central follicular lymphoma (Reed et al., 1988). Elevated quantities of Bcl-2, induced by the promoter of the light chain immunoglobulin, play a role in bestowing confrontation to apoptosis upon converted B cells. Bcl-2 is detectable in conjunction with several cellular components, encompassing the nuclear membrane, endoplasmic reticulum, and the outer mitochondrial membrane (Popgeorgiev et al., 2018). Moreover, within the extensive Bcl-2 protein family, there exist several other anti-apoptotic members, such as Bcl-xL, Bcl-W, Mcl-1, and A1. Additionally, there are pro-apoptotic proteins known as BH3-only proteins, which include Bax and Bak. These proapoptotic proteins have the ability to induce permeability transition in the mitochondrial membrane by forming pores. Collectively, all of these proteins constitute the broader Bcl-2 protein family (Willis et al., 2005; Lomonosova and Chinnadurai, 2008). Bax and Bak, and occasionally Bok, are effector molecules with the ability to change their conformation, associate with each other to form oligomers, and generate pores within the outer mitochondrial membrane. This sequence of events ultimately results in the permeability transition within the mitochondria (Cosentino and García-Sáez, 2017). As pores are formed in the outer mitochondrial membrane, simultaneous modifications occur in the structure of the inner membrane, along with a restructuring of cristae. These modifications facilitate the movement of proteins from the space between the mitochondrial membranes (intermembrane space) into the cytosol.

There is an alternative hypothesis proposing that the Bcl-2 protein family might play a role in the regulation of an existing multi-protein complex recognized as the permeability transition pore (PTP). This complex consists of a combination of soluble and integral proteins found within the mitochondrial membranes (Brenner et al., 2000; Azzolin et al., 2010). It’s important to emphasize that almost all proteins comprising the mitochondrial permeability transition pore (MPTP) come in multiple forms, suggesting that their expression profiles could differ between normal and transformed cells. This pore complex is situated at specific points where the inner and outer mitochondrial membranes make contact. When the pore transitions into an “open” state, it leads to the expanding of the matrix and disruption of the membrane (Hurst et al., 2017). There is supporting evidence indicating that the formation of the mitochondrial permeability transition pore (MPTP) can play a role in post-apoptotic necrosis. Moreover, it can be initiated by the presence and actions of the p53 protein (Ying and Padanilam, 2016). When exposed to irradiation by UV and various stimuli, cytosolic p53 can relocate toward the outer membrane of mitochondria. There, it straightforwardly reacts with Bcl-2 proteins. This interaction can bind to both the antiapoptotic protein Bcl-xL and the proapoptotic proteins Bax and Bak. Consequently, this binding event triggers the generation of mitochondrial outer membrane permeabilization (MOMP), which subsequently leads to the activation of the caspase cascade (Qian et al., 2022).

Orexins induce the activation of certain tyrosine phosphorylation events in the OX1R receptor, targeting specific tyrosine-based patterns referred to as the immunoreceptor tyrosine-based inhibitory motif (ITIM) and the immunoreceptor tyrosine-based switch motif (ITSM) (El Firar et al., 2009). Due to these phosphorylation processes, it leads to the recruitment and activation of the phosphotyrosine phosphatase SHP-2 (Voisin et al., 2008). Consequently, this activation initiates mitochondrial apoptosis mediated by cytochrome c (Marcos and Coveñas, 2023).

In contrast, the administration of orexin A was observed to block the movement of cytochrome c from the mitochondria to the cytoplasm and its binding with Apaf-1 (Danial and Korsmeyer, 2004; Johnson et al., 2007; Xu et al., 2021b). Consequently, this interference would result in a decrease in the mobilization of procaspase 9, as shown in Table 2. Following treatment with orexin A, there was an observed increase in the expression of Bcl-2, accompanied by a decrease in the expression of Bax. Therefore, this led to an elevation in the Bcl-2/Bax ratio (Safdar et al., 2021). Furthermore, in the context of cerebral ischemia/reperfusion injury, orexin A demonstrated the inhibition of cytochrome C expression, as well as the cleaved forms of caspase-9 and caspase-3 (Xu et al., 2021b). As a result, it can be inferred that orexin A mitigates apoptosis by modulating the Bcl-2/Bax ratio and suppressing the expression of cytochrome C, cleaved caspase-9, and cleaved caspase-3. Orexin A also demonstrated a neuroprotective effect against cellular death induced by palmitic acid, as shown in Table 2. Specifically, orexin A attenuates palmitic acid-induced cell death in the hypothalamus through mechanisms that include a reduction in caspase-3/7-driven apoptosis, the Bcl-2 gene expression stabilization, and a reduction in the Bax/Bcl-2 expression ratio (Duffy et al., 2016). The intraperitoneal administration of orexin A resulted in the improvement of renal function and the alleviation of histological abnormalities in mice treated with cisplatin. Orexin A achieved this by curbing cisplatin-induced oxidative stress and decreasing apoptotic cell death through the inhibition of the p53-mediated pathway in these cisplatin-treated mice (Jo et al., 2022). Furthermore, when OXR1 was blocked in testicular cancer in mice, there was a notable increase in the expression levels of Bax, p53, and caspase 3. This observation implies that the activation of OXR1 potentially results in a reduction in apoptosis by inhibiting the p53/caspase 3 signaling pathway.

## 8 Effect of orexins on receptor-related mechanism of apoptosis

Substances that trigger apoptosis are categorized as ligands within the cytokine group known as the TNF superfamily. These ligands specifically engage with death receptors found within the TNF-R superfamily (Diaz Arguello and Haisma, 2021). These cytokines possess the ability to attach to a varied range of surface receptors, and as of now, more than 40 pairs of ligands and receptors have been recognized within this particular family (Aggarwal et al., 2012). The TNF superfamily consists of multiple members, among which are APRIL, TRAIL, TNF-α, FasL, RANKL, TWEAK, and lymphotoxinα (Devarapu et al., 2017). For instance, FasL demonstrates a unique binding preference for the Fas receptor, whereas TRAIL possesses the capability to engage with four separate receptors referred to as TRAIL-R1 through TRAIL-R4 (Diaz Arguello and Haisma, 2021). When the TNF-R superfamily is activated, its intracellular domain initiates the recruitment of adaptor proteins. For instance, in the case of Fas receptor activation by FasL, it associates with adaptor proteins such as FADD/MORT1. Subsequently, these adaptor proteins prompt the activation of procaspases 8 or 10 (Diaz Arguello and Haisma, 2021). Following this, these procaspases undergo maturation into their active states, and these active caspases subsequently trigger the activation of executive caspases. For instance, caspase 8 has the capacity to activate Bax, thus playing a role in apoptosis through the mitochondrial pathway (Diaz Arguello and Haisma, 2021).

The TNFR1 receptor can assemble into two distinct complexes that involve various adaptor proteins: complex I and complex II. Complex I comprises adaptor molecules such as TRADD, RIPK1, TRAFs, cIAP1 or 2, and cFLIP (Dondelinger et al., 2016). The activation of RIPK1 leads to the creation of a platform that initiates the activation of NF-κB and MAPK signaling pathways. This activation subsequently triggers the expression of prosurvival proteins and proinflammatory factors (Chen et al., 2019). Complex II, on the other hand, involves TRADD-adaptor proteins interacting with FADD-adaptor proteins, ultimately resulting in apoptosis. However, if the activation of TNF-R1 results in the degradation of cIAPs and inhibition of caspase activity via the RIPK1-mediated signaling, it prompts another cell death type called necroptosis (programmed necrosis) (Liu et al., 2016). Necroptosis is induced by various factors, including but not limited to TNFα, Fas, interferons, TRAIL/Apo2L, viral infections, and toll-like receptors (Jayaraman et al., 2021).

It is interesting to observe that besides OX1R, which initiates apoptosis through the intrinsic or mitochondrial pathway, it might modulate receptor-mediated mechanism of apoptosis (Voisin et al., 2011, 2022). Cancer cells are also known to express Fas receptors, which, acting as death receptors, initiate apoptosis through the extrinsic apoptosis pathway (Dondelinger et al., 2016). Regrettably, many cancer cell lines tend to exhibit some degree of resistance to Fas ligand-mediated apoptosis, even when they possess Fas receptors on their surface (Zhu et al., 2017). In contrast, it’s worth noting that normal cells are highly sensitive to Fas-mediated apoptosis, responding with a high degree of susceptibility to this signaling pathway (Green and Ferguson, 2001). This significant difference in sensitivity between cancer cells and normal cells poses a substantial obstacle to the potential use of Fas receptor agonists as candidates for chemotherapy. If these agonists were employed, patients’ cancer cells might remain relatively resistant to apoptosis, while their normal cells would be more susceptible to self-destruction. Similarly, resistance to apoptosis facilitated by TNF-related apoptosis ligand (TRAIL) has been observed at multiple points in the extrinsic apoptosis pathway in cancer, hindering the therapeutic utility of TRAIL as an apoptosis inducer in tumor cells. Efforts to utilize TNF and Fas ligand have also faced challenges due to the induction of NF-κB-mediated inflammation (Rex et al., 2019) and fulminant hepatic failure, respectively, (De Gregorio et al., 2020). Notably OX1R is evidently unaffected by the limitations typically associated with death receptors. Notably, OX1R induces apoptosis in colon cancer cells, even those resistant to 5-FU, as reported by Voisin and colleagues (Voisin et al., 2011, 2022). This unique characteristic positions OX1R as a promising candidate for therapeutic intervention, with OX1R agonists potentially enhancing the efficacy of traditional chemotherapy treatments in colon cancer (Wolpin and Mayer, 2008; Segal and Saltz, 2009).

Some studies stated that administration of orexin A reduced the formation of TNF-α and other cytokines and led to anti-inflammatory and antiapoptotic effects in colitis (Messal et al., 2018) (Table 2). This could be mediated by inhibition of NF-κB and MAPK/ERK signaling (Xu et al., 2021b). However, Excessive release of TNFα leads to a downregulation of OX2R as previously reported (Sun et al., 2018). The administration of TNFα significantly decreased (by 86%) OX2R protein levels through ubiquitination in B35 cells which was accompanied by a diminution in cIAP-1 and 2 levels (Zhan et al., 2011). These research studies revealed that TNF-α can impede the function of the orexin system by decreasing the levels of both Prepro-orexin and OX2R (Zhan et al., 2011). However, further studies are needed to investigate the effect of orexins on the TNF superfamily and their adaptor proteins.

## 9 Endoplasmic reticulum stress and apoptosis

The Endoplasmic Reticulum (ER) possesses a protein quality management system that depends on the assistance of protein chaperones and foldases. These components play a critical role in recognizing and facilitating the correct folding of misfolded proteins. If the process of folding is unsuccessful, it leads to the buildup of proteins that are misfolded within the lumen of ER, a condition referred to as ER stress (Haeri and Knox, 2012). These issues encompass problems like impaired glycosylation, reduced formation of disulfide bonds, a reduction in the concentration of calcium within the ER lumen, and disruptions in the transport of protein to the Golgi apparatus. The culmination of these events triggers the Unfolded Protein Response (UPR) signaling pathway, which is designed to either destroy or correct misfolded proteins and restore equilibrium within the Endoplasmic Reticulum (ER) (Amen et al., 2019). The UPR can initiate either an adaptive response or a reaction that results in apoptotic cell death. Following the ER stress onset, cells can activate several mechanisms, including: (1) reduced protein synthesis to prevent further accumulation of proteins (Almanza et al., 2019), (2) Enhanced gene transcription responsible for chaperone proteins encoding for instance GRP94 and BiP/GRP78 (Zhu and Lee, 2015), and (3) ER-accompanying degradation (ERAD) of proteins that fail to embrace their intrinsic conformation, thereby targeting them for disposal (Qi et al., 2017). Degradation can take place via autophagy or within the 26S proteasome. In mammals, 3 transmembrane proteins, specifically IRE-1α, PERK, and ATF-6α, typically associate with GRP78, a chaperone responsible for facilitating proper protein folding and preventing protein aggregation (Bravo et al., 2013). In cases of mild or transient ER stress, GRP78 separates from IRE-1, ATF-6α, and, PERK, triggering the unfolded protein response (UPR) to mitigate stress and uphold ER functionality (Wang et al., 2015). When the cell’s adaptive reaction to ER stress falls short, programmed cell death is initiated through multiple mechanisms. These mechanisms encompass the IRE1-activated ASK1/JNK signaling, the PERK/eIF2-supported activation of the proapoptotic transcription factor CHOP, Bax/Bak-mediated calcium release into the cytosol, and the proteolytic stimulation of procaspase-12 (Merighi and Lossi, 2022). IRE1α stands out as one of the highly conserved sensors for ER stress. It’s categorized as a type I transmembrane protein with dual functionalities, serving as both an endoribonuclease and a serine/threonine kinase. When GRP78 disengages, IRE1α experiences homo-oligomerization and trans-autophosphorylation events, which then activate its endoribonuclease and kinase domains (Ricci et al., 2021). As a result of this activation, IRE1α becomes engaged in facilitating the splicing of the mRNA intron responsible for encoding XBP1. This splicing process yields the XBP1 isoform (active), which subsequently initiates the gene expression responsible for ensuring the accurate folding of proteins within the lumen, the phospholipids biosynthesis, and the degradation of misfolded molecular structures (Yoshida et al., 2001). IRE1α also plays a role in engaging signaling pathways that lead to apoptotic cell death. For instance, it interacts with TRAF2 in the cytosol, leading to the activation of NF-κB, ASK, and JNK, ultimately contributing to inflammation and apoptosis (Nishitoh et al., 2002; Tam et al., 2012; Junjappa et al., 2018). IRE1α has the capacity to interact with proapoptotic proteins like Bax and Bak. This interaction induces conformational changes and/or oligomerization within the ER membrane, resulting in the formation of a pore that permits the secretion of calcium into the cytosol (Kanekura et al., 2015). The heightened calcium concentration activates procaspase-12. Subsequently, procaspase-12 dissociates from the membrane, arrives the cytosol, and triggers the stimulation of procaspase-9, ultimately leading to the activation of caspase-3 (Moon, 2023). The calcium that entered the cytosol from the ER is promptly sequestered by the mitochondria. This event results in the depolarization of the inner mitochondrial membrane and initiates mitochondria-supported apoptosis.

PERK is a transmembrane sensor protein equipped with serine/threonine kinase activity within its cytoplasmic domain. When the endoplasmic reticulum (ER) experiences stress, PERK becomes active in a manner similar to IRE1. This activation process entails the separation of IRE1α from GRP78, its subsequent homooligomerization, and autophosphorylation (Rozpedek et al., 2016). After activation, active PERK phosphorylates the α-subunit of eIF2α (eukaryotic initiation factor 2α), which leads to a decrease in general protein synthesis. Paradoxically, this also results in an elevation in the translation of specific mRNAs, including those responsible for encoding the transcription factor ATF4 (Rozpedek et al., 2016). ATF4 plays a pivotal role in the cellular response to endoplasmic reticulum (ER) stress. It initiates the expression of other unfolded protein response (UPR) transcription factors, chaperones, proteins involved in autophagy regulation, and components of the oxidative stress response. One significant target of ATF4 is the gene responsible for producing the proapoptotic protein CHOP. CHOP, in turn, enhances the transcription of oxidase ERO1α, which results in the generation of reactive oxygen species (ROS) within the ER. Additionally, it triggers the release of calcium ions (Ca2+) into the cytosol by activating the inositol 1,4,5-trisphosphate receptor located on the ER membrane (Cao and Kaufman, 2014). The increased Ca2+ levels activate Ca2+/calmodulin-dependent protein kinase II (CaMKII), which, in turn, initiates apoptosis. CHOP further promotes apoptosis by inhibiting the antiapoptotic protein Bcl-2 and activating other proapoptotic factors. Additionally, it plays a role in the activation of caspase-8, further contributing to the apoptotic process (Hu et al., 2018).

Intervention with orexin A led to a substantial reduction in the expression of GRP78 and protected against cell damage induced by oxygen–glucose deprivation/reoxygenation (Kong et al., 2019). Orexin A exhibited an anti-apoptotic effect by diminishing the expression of components within the ER stress-related signaling pathways, including GRP78/IRE1α/JNK and GRP78/IRE1α/caspase-3/caspase-12 (Xu et al., 2021a), which provided new mechanistic evidence of the neuroprotective effect of orexin-A. Furthermore, treatment with orexin-A led to a reduction in the infarct volume in rats following cerebral ischemia–reperfusion injury, while also mitigating neuronal apoptosis (Xu et al., 2021a). Taken together, these discoveries imply that orexin-A has the potential to alleviate cerebral ischemia–reperfusion injury by inhibiting apoptosis mediated by ERS, thereby elucidating the mechanism underlying its neuroprotective properties (Table 2).

It has been observed that the administration of orexin-A can prevent the development of non-alcoholic steatohepatitis. Notably, orexin A promptly activates the mTOR-XBP1 pathway in the liver during periods of fasting (Tsuneki et al., 2022). The daily administration of orexin A was found to effectively reduce hepatic inflammation during the refeeding phase following fasting, and this effect was dependent on the presence of rapamycin. Consequently, it was proposed that orexin’s action could potentially prepare the liver’s adaptive response to anticipated endoplasmic reticulum (ER) stress through the mTOR/XBP1 pathway, thereby preventing an excessive occurrence of ER stress and inflammation in the liver (Tsuneki et al., 2022). Furthermore, a deficiency of orexin A in the hypothalamus could result in neuronal apoptosis, a process mediated by pathways that encompass IRE1α-XBP1, PERK, and unfolded proteins.

In response to stimulation by orexin-A, a common cellular reaction is the elevation of intracellular calcium ion (Ca2+) levels. Research has demonstrated that orexins induce intracellular Ca2+ transients in cells expressing the orexin receptors, whether they are receptor-transfected cells or cells naturally expressing these receptors. These Ca2+ transients serve as valuable indicators of orexin receptor activation (Kukkonen and Leonard, 2014). Typically, increases in intracellular Ca2+ concentration can result from various mechanisms. Studies conducted in Chinese hamster ovary (CHO) cells indicate that the origin of orexin-A-induced Ca2+ transients varies depending on the concentration of the ligand (Smart et al., 1999). At lower concentrations of orexin-A, the primary source of Ca2+ influx appears to be the opening of receptor-operated Ca2+ channels. However, at higher concentrations of orexin-A, it is suggested that the increases in Ca2+ are a consequence of both Ca2+ release from the endoplasmic reticulum and influx through store-operated Ca2+ channels. This concentration-dependent variation in the mechanism of Ca2+ entry points to a complex and regulated cellular response to orexin-A stimulation (Xia et al., 2009). Indeed, an excessive accumulation of Ca2+ or disruptions in the precise compartmentalization of intracellular calcium can contribute to the initiation of apoptosis. Proper regulation of intracellular calcium levels is essential for cell survival, and dysregulation can lead to cell death pathways, including apoptosis (Orrenius et al., 2003). Exactly, the apoptotic effect of orexin mediated by Ca2+ is contingent upon the intracellular concentration of calcium. The level of intracellular calcium plays a pivotal role in determining whether cells undergo apoptosis or not, and it can be influenced by various factors, including the actions of orexin (Table 2).

## 10 Effect of orexins on nuclear mechanisms of apoptosis

The process of nuclear-mediated apoptosis encompasses two primary pathways. The first pathway is associated with the activation of p53, which subsequently upholds the expression of proapoptotic proteins, including Noxa, Puma, and Bax (Oda et al., 2000; Nakano and Vousden, 2001; Chen L. et al., 2013). These proapoptotic proteins, once expressed, contribute to mitochondrial-driven apoptosis and initiate the caspase cascade. The activation of p53 can be triggered by various factors, including exposure to ionizing radiation and UV, heat shock, hypoxia, oxidative stress, and reduced temperatures (Renzing et al., 1996). In actively growing cells, p53 activation can result from DNA damage or errors in DNA replication. This leads to cell cycle arrest, a process mediated by the expression of the p21 gene product under the control of p53. If the cellular repair mechanisms are unable to effectively address the damage, the cells will then initiate their apoptotic program as a safeguard against potential genetic instability (Williams and Schumacher, 2016).

The second pathway for inducing nuclear apoptosis involves the activation of caspase-2. Despite much research into its role in apoptosis, the function of caspase-2 remains unclear. Caspase-2 has a similar domain structure to initiator caspases-8 and -9 and becomes active through dimerization and autoprocessing (Bouchier-Hayes and Green, 2012). It comprises a higher MW protein platform featuring a CARD sequence that participates in protein–protein interactions. Specifically, the CARD in caspase-2 interacts with the CARD in RAIDD, functioning as an adaptor protein that facilitates the recruitment of caspase-2 into the activation complex (Vigneswara and Ahmed, 2020).

The protein known as “p53-induced protein with a death domain” (PIDD) is triggered by p53 and plays a role in p53-dependent apoptosis. The assembly of the PIDDosome, a complex comprising PIDD, RAIDD, and caspase-2, occurs when PIDD binds to RAIDD, which in turn recruits seven caspase-2 molecules. This recruitment leads to autoprocessing and activation of caspase-2 within the PIDDosome complex (Sladky and Villunger, 2020). PIDD also forms a separate complex with RIPK1 and NEMO in response to DNA damage (Weiler et al., 2022). The recruitment of RAIDD and RIPK1 into the PIDDosome occurs sequentially, with RIPK1 and NEMO first interacting with PIDD, followed by RAIDD and caspase-2 (Table 2).

There have been numerous studies investigating the effect of orexin on the p53 protein, a transcription factor that plays a crucial role in cell cycle regulation and DNA damage response. In *in vitro* studies, orexin has been shown to regulate the activity of p53, leading to changes in cell proliferation, apoptosis, and senescence. For example, orexin treatment has been reported to induce p53-mediated apoptosis in various cancer cell lines (Ammoun et al., 2006), including prostate (Malendowicz et al., 2011), and colon (Wen et al., 2016) cancer cells.

Conversely, orexin has also been shown to protect neurons against oxidative stress-induced apoptosis by stabilizing the p53 protein. *In vivo* research has further confirmed the role of orexin in p53 regulation. For example, a study in mice showed that orexin deficiency leads to increased p53 accumulation and apoptosis in the hypothalamus, suggesting a potential role for orexin in maintaining hypothalamic neuronal viability (Chen et al., 2015). Another study demonstrated that orexin receptor agonists can protect from oxidative stress-induced p53 activation and apoptosis in neuronal cell cultures (Sokołowska et al., 2014).

Recent research showed that orexin may also affect caspase-2. One study found that orexin reduced caspase-2 activity in neurons, suggesting a potential neuroprotective effect (Chung et al., 2007). This was further supported by a study, which showed that orexin treatment significantly reduced caspase-2-mediated cell death in a model of cerebral ischemia (Chen et al., 2011). A more recent study found that orexin-A could protect neurons against oxidative stress-induced cell death by regulating the balance between the pro-apoptotic protein, caspase-2, and the anti-apoptotic protein, Bcl-2. The study suggested that orexin-A may play a protective role in some neurodegenerative diseases by reducing oxidative stress and inhibiting the activation of caspase-2 (Naidoo, 2022).

## 11 Conclusion

In conclusion, the multifaceted effects of orexins on apoptosis highlight their complex and context-dependent role in cellular regulation. The evidence presented in this text demonstrates that orexins can modulate apoptosis through various mechanisms, including caspase activation, mitochondrial regulation, receptor-related pathways, endoplasmic reticulum stress, and nuclear mechanisms.

**Figure 3:**
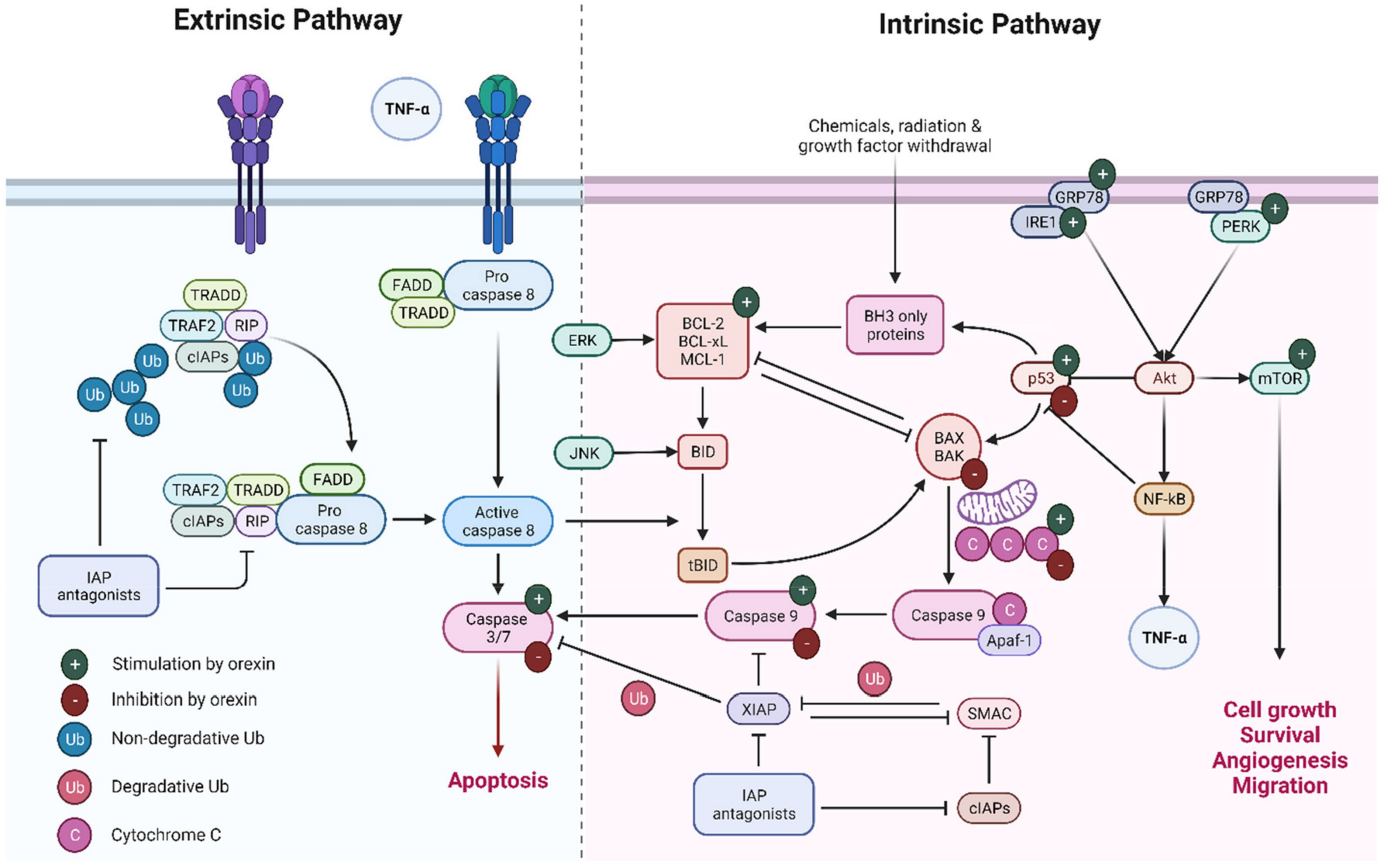
Extrinsic and intrinsic pathways of apoptosis.

Orexins exhibit both pro-apoptotic and anti-apoptotic properties, depending on the cell type and conditions. In cancer models, orexins have been shown to promote apoptosis via the stimulation of caspases, particularly caspase-3 and caspase-9, in several cell lines. This pro-apoptotic effect has potential implications for cancer therapy, as it selectively targets cancer cells while sparing normal cells. Conversely, orexins also demonstrate anti-apoptotic effects by inhibiting caspase activity, particularly caspase-3, in neuronal cells. These effects contribute to neuroprotection against oxidative stress-induced apoptosis, which may have relevance in the context of neurodegenerative diseases. Furthermore, orexins influence apoptosis through mechanisms such as the regulation of Bcl-2/Bax ratio, cytochrome C expression, and modulation of p53 activity. These effects are intricate and contingent on the specific cellular and physiological contexts. The possible effects of orexin on different molecular mechanisms of apoptosis are summarized in Figure 3.

In summary, orexins play a pivotal role in the intricate regulation of apoptosis, with their actions varying depending on the cellular environment and signaling pathways involved. Understanding these diverse effects of orexins on apoptosis is crucial for elucidating their potential therapeutic applications in cancer treatment and neuroprotection.

## 12 Future perspectives

Further research is needed to fully understand the mechanisms by which orexins regulate apoptosis. The following areas could be explored: (1) Additional studies are needed to investigate the effects of orexins on apoptosis in different cell types and to determine whether the conflicting findings in the literature are due to differences in cell type; (2) More investigations are needed to understand the underlying mechanisms by which orexins regulate apoptosis, including the role of intracellular Ca2+ concentration, the p53 protein, caspases, and other signaling pathways; (3) The relationship between orexins and oxidative stress is complex and not fully understood. Supplementary research is required to clarify the role of orexins in reducing oxidative stress and its impact on apoptosis regulation; (4) Orexins have been shown to have neuroprotective effects in various contexts, but the underlying mechanisms are not well understood. Therefore, it is needed to determine the precise mechanisms by which orexins protect against neuronal injury and death; (5) The role of orexins in cancer is not well understood and requires further investigation to determine whether orexins can be used as therapeutic targets in cancer treatment and to clarify the factors that could determine their role as both pro- and anti-apoptotic agents in cancer cells.

In the domain of intrinsic apoptotic mechanisms, orexin is implicated in initiating a series of molecular events that culminate in apoptosis, commencing with the activation of p53. This crucial step leads to the mitochondrial release of cytochrome c, which subsequently activates caspase 9, an essential enzyme in the apoptotic process. Concurrently, orexin modulates the Akt pathway by upregulating key proteins involved in cellular stress responses. This modulation results in the activation of several critical factors that significantly influence apoptosis and cell survival. NF-κB plays a dual role, predominantly acting as an anti-apoptotic agent by downregulating p53 expression, while mTOR promotes cell survival, migration, and angiogenesis. Interestingly, under certain conditions, orexin appears to downregulate the p53/cytochrome c/caspase 9 axis, thereby inhibiting apoptosis. In the context of extrinsic apoptotic mechanisms, orexin’s influence on caspase 3 and caspase 7 is context-dependent, with the potential to either stimulate or inhibit these enzymes. In various cancer models, orexins increased apoptosis via induction of caspase 3 and 7. In chemoresistant pancreatic tumors, orexins effectively inhibit tumor development by enhancing caspase 3 and 7-mediated apoptosis. Additionally, orexins exhibit a contrasting role in neuronal cells by inhibiting caspase-3 activity and protecting against apoptosis.

## Author contributions

SC: Conceptualization, Data curation, Investigation, Project administration, Software, Supervision, Validation, Writing – original draft, Writing – review & editing. SS: Conceptualization, Data curation, Investigation, Project administration, Software, Writing – original draft, Writing – review & editing. RH: Conceptualization, Data curation, Funding acquisition, Investigation, Methodology, Visualization, Writing – original draft, Writing – review & editing. MA-R: Conceptualization, Data curation, Investigation, Software, Validation, Writing – original draft, Writing – review & editing. MY: Conceptualization, Data curation, Investigation, Methodology, Project administration, Software, Supervision, Validation, Visualization, Writing – original draft, Writing – review & editing. EE: Conceptualization, Data curation, Methodology, Formal analysis, Validation, Investigation, Writing – original draft, Writing – review & editing.

## Glossary

AgRP: Agouti-related peptide
Aprf-1: Apoptotic protease activating factor 1
APRIL: Proliferation-inducing ligand
Bax: Bcl-2-associated X protein
Bcl-2: B-cell lymphoma 2
cAMP: Cyclic adenosine monophosphate
FADD: Fas-associated death domain protein
Fas: Cell surface death receptor
FasL: Fas ligand
FLICE: (FADD-like IL-1β-converting enzyme)-inhibitory protein
GPCRs: G-protein-coupled receptors
LC: Locus coeruleus
LH: Lateral hypothalamic
MAPK: Mitogen-activated protein kinase
MOMP: Mitochondrial outer membrane permeabilization
MORT1: Mediator of receptor-induced toxicity protein 1
MPTP: Mitochondrial PTP
NF-κB: Nuclear factor-kappa B
NMR: Nuclear magnetic resonance
NYP: Neuropeptide Y
OX1R: Orexin-1 receptor
OX2R: Orexin-2 receptor
PFA: Perifornical hypothalamic areas
PLC: Phospholipase C
